# Effect of bone marrow stromal cell transplantation on neurologic function and expression of VEGF in rats with focal cerebral ischemia

**DOI:** 10.3892/mmr.2014.2502

**Published:** 2014-08-20

**Authors:** NAN LI, PING WANG, XUE-LING MA, JUAN WANG, LI-JING ZHAO, LI DU, LI-YA WANG, XIN-RUI WANG, KANG-DING LIU

**Affiliations:** 1 Department of Neurology, The First Bethune Hospital of Jilin University, Changchun, Jilin 130021, P.R. China; 2 Neonatal Division of the Pediatric Department, The First Bethune Hospital of Jilin University, Changchun, Jilin 130021, P.R. China; 3 Department of Neurology, The Fourth Hospital of Harbin Medical University, Harbin, Heilongjiang 150001, P.R. China; 4 The Otolaryngology Research Institute, The First Bethune Hospital of Jilin University, Changchun, Jilin 130021, P.R. China; 5 Amphixenosis Research Institute, Jilin University, Changchun, Jilin 130000, P.R. China

**Keywords:** bone marrow stromal cells, middle cerebral artery occlusion, vascular endothelial growth factor, stem cell therapy, neurological severity score

## Abstract

There is evidence that the transplantation of mesenchymal stem cells into rat models of cerebral ischemia reduces ischemic damage; however, the mechanism remains to be elucidated. The present study aimed to assess the effect of transplantation of human bone marrow stromal cells (hBMSCs) on neurologic function and the expression of vascular endothelial growth factor (VEGF) in a rat model of focal cerebral ischemia. The left middle cerebral artery of adult Wistar rats was occluded for 90 min using a nylon thread, followed by reperfusion for 1 h. hBMSCs labeled with 5-bromo-2-deoxyuridine (BrdU) were stereotaxically injected into the ischemic boundary zone. Behavioral analysis using the Neurological Severity Score (NSS) was conducted on days 1, 3, 7 and 28, and a histologic evaluation was performed simultaneously. VEGF was detected by immunofluorescence staining and western blot analysis. Fifty rats were divided equally into five groups: Normal control, sham-operated, operated (no transplantation), Dulbecco’s medium Eagle’s medium (DMEM)-injected (received only serum-free DMEM), and hBMSC-transplanted. The hBMSC-transplanted group showed significantly improved behavioral recovery compared with the operated and DMEM-transplanted groups on days 3, 7 and 28. Histological examination showed that transplanted cells migrated from the injection site into nearby areas including the cortex. Expression of VEGF was significantly greater in the hBMSC group compared with the other four groups on each assessment day. The expression of VEGF was found to be beneficial for functional recovery following cerebral ischemic injury and hBMSC transplantation stimulated the expression of VEGF. Transplantation of BMSCs may be a promising therapeutic strategy for treating cerebral infarction.

## Introduction

Although intrinsic progenitor cell proliferation and differentiation occur in response to cerebral ischemia in order to provide functional benefits, this is not sufficient to prevent irreversible brain damage. The transplantation of neural stem cells (NSCs) into animal models of cerebral ischemia is able to produce functional recovery; however, these cells are only available in the fetal forebrain or the subventricular zone of the adult brain, which raises ethical concerns and therefore, there is an inadequate supply for therapeutic use (1). Bone marrow stromal cells (BMSCs) have attracted interest as a promising alternative for clinical application as they are easy to isolate from bone marrow and are readily expanded in number *in vitro* (2). They may also be used for autotransplantation without ethical problems or inducing any immune response.

A number of studies have shown that BMSC transplantation is able to improve functional recovery following cerebral ischemia (3–10). However, the mechanisms by which BMSCs promote functional recovery remain to be elucidated. It has been proposed that BMSCs secrete several trophic factors or stimulate the host brain to express trophic factors (11–13). Trophic factors that have been reported to be secreted by mesenchymal stem cells include vascular endothelial growth factor (VEGF), basic fibroblast growth factor (bFGF), insulin-like growth factor 1 (IGF-1), neurotrophin-3 (NT-3), and brain-derived neurotrophic factor (BDNF) (4). VEGF promotes angiogenesis as well as neurogenesis (4). A stroke is able to induce angiogenesis, which is associated with improved neurologic recovery; however, under normal circumstances, angiogenesis induced by stroke is insufficient for functional recovery. Since BMSCs secrete VEGF, the secretion of this trophic factor by BMSCs or the stimulation of host cells by BMSCs to secrete VEGF may have an important role in the improvement of cerebral ischemia observed following BMSC transplantation. The aim of the present study was to investigate the changes that occur over time in a rat model of middle cerebral artery occlusion (MCAO) when human BMSCs (hBMSCs) are transplanted into the brain following reperfusion with a particular focus on the expression of VEGF. hBMSCs were selected for use in the present study as they are relevant to clinical practice in humans and as transplantation of hBMSCs into rodents has been demonstrated to be histocompatible (14,15).

## Materials and methods

Experimental procedures were approved by the Animal Experimentation Ethics Committee of the Medical College of Jilin University (Changchun, China).

### Culturing of hBMSCs

hBMSCs were isolated from healthy adult human ilium bone marrow and incubated in low-glucose Dulbecco’s modified Eagle’s medium (L-DMEM; Gibco, Life Technologies, Carlsbad, CA, USA). Cells subcultured four times were characterized by flow cytometry for CD34, CD45, CD29, and CD44 (R&D Systems, Inc., Minneapolis, MN, USA), and then co-cultured (4×10^5^ cells/μl) with 5-bromo-2-deoxyuridine (BrdU; Sigma-Aldrich, St. Louis, MO, USA) for 48 h prior to transplantation. The total number of transplanted cells was 2×10^6^ cells in 5 μl medium in accordance with previous studies (14–17).

### Middle cerebral artery occlusion rat model

Male Wistar rats (Charles River Laboratories, Beijing, China) weighing 250–280 g were kept at room temperature (24°C) with a 12-h light-dark cycle and were given free access to food and water. The MCAO procedure was a modification of the methods described by Koizumi *et al* (18) and Longa *et al* (19). Briefly, under deep anesthesia induced by 10% chloral hydrate (0.3 ml/100 g; Sigma-Aldrich), a midline cervical incision was made and the left carotid bifurcation was identified and exposed. A probe made of nylon thread with a diameter of 0.225 mm was inserted into the ligated external carotid artery and advanced into the internal carotid artery to a position 18±0.5 mm from the bifurcation. Following 90 min of ischemia, the suture was slightly withdrawn for reperfusion for 1 h. During the surgery, the rectal temperature was maintained between 37.5 and 38°C using a feedback heating pad through all the surgical and postoperative procedures until the rats regained consciousness. The sham rats were subjected to exposure of the internal carotid artery (ICA) and external carotid artery, and the control rats were not treated.

### Transplantation

One hour following MCAO, the rats were anesthetized by intraperitoneal injection of 10% chloral hydrate (0.3 ml/100 g) and placed onto a stereotaxic frame. A suspension of 2×10^6^ cells in 5 μl L-DMEM (Gibco, Life Technologies) was stereotaxically injected into the ischemic boundary zone from 3 mm anterior to the bregma and 1 mm lateral to the midline. Injections were given 4 mm below the cortical surface in each case. Five groups of animals were prepared: i), Normal control group, which did not receive any surgery (n=10); ii) sham-operated group, in which a midline cervical incision was made and the right carotid bifurcation was identified and exposed, but no probe was inserted (n=10); iii) operated group, which received no transplantation following ischemia/reperfusion (n=10); iv) DMEM-injected group, which received only serum-free DMEM after ischemia/reperfusion (n=10); v) hBMSC-transplantated group, which underwent transplantation of BMSCs following ischemia/reperfusion (n=10).

### Behavioral assessment

The severity of neurological damage was evaluated using Chen’s Neurological Severity Score (NSS) system (Table I) (5). Neurologic function was graded on a scale of 0–18 (0, normal score; 18, maximal deficit score). The NSS is a composite of motoric, sensory, reflex, and balance assessments. In the severity score of injury, one point was awarded for the inability to perform a task or for the lack of an assessed reflex; thus, the more severe the injury, the higher the score. The recovery of neurologic function was observed and the scores of all rats were recorded on days 1, 3, 7, and 28 following transplantation.

### Histological analysis

Rats from each group were sacrificed following each behavioral assessment session to examine the existence and distribution of human BMSCs and the expression of VEGF in the brain tissue. The rats were sacrificed by administration of an overdose of 10% chloral hydrate (Sigma-Aldrich) and then transcardially perfused with 0.9% saline followed by 4% paraformaldehyde fixative solution (Sigma-Aldrich). The brain was embedded in paraffin and cut into coronal blocks of 5 μm using a brain slicer (Zivic Brain Matrix; Zivic Instruments, Pittsburgh, PA, USA). For immunofluorescence staining, sections were incubated with a mouse monoclonal primary antibody to BrdU (1:100; Santa Cruz Biotechnology, Inc., Santa Cruz, CA, USA) or a rabbit polyclonal primary antibody to VEGF (1:100; Santa Cruz Biotechnology, Inc.) at 4°C overnight. Following three washes with phosphate-buffered saline (PBS), sections were incubated with an Alexa Fluor 488-labeled goat anti-mouse immunoglobuiln G (IgG) secondary antibody (1:400; Zhongshan Belling Biotechnology, Guangdong, China) or an Alexa Fluor 555-labeled goat anti-rabbit IgG secondary antibody (1:400; Zhongshan Belling Biotechnology) for 1 h at room temperature in the dark. Following washing with PBS, Hoechst 33342 (R&D Systems, Inc.) was used for nuclear staining and the sections were mounted with glycerol and examined using fluorescence microscopy. Five sequential slides from each block were examined (magnification, ×200) and the number of VEGF-positive cells in each slide was counted. The mean number of positive cells from the five slides was recorded. This was considered to be representative of the total number of VEGF-positive cells in the whole brain. BrdU-positive cells were also observed using fluorescence microscopy.

### Western blot analysis

Rats from each group were sacrificed and perfused as described above. Brains were dissected and frozen at −70°C. Western blot analysis was performed using a primary polyclonal rabbit anti-rat VEGF antibody (1:100; Santa Cruz Biotechnology) and a secondary goat anti-rabbit IgG antibody (1:400 dilution; Zhongshan Belling Biotechnology). The primary antibody used binds to the amino acid splice variants 189, 165 and 121 of VEGF.

### Statistical analysis

Data are presented as the mean ± standard deviation (SD). One-way analysis of variance (ANOVA) with the Bonferroni post-hoc test was performed to compare the difference between groups at each time point. Statistical assessments were all two-sided, and the statistical significance level was set as P<0.05. Statistical analyses were performed using SPSS 15.0 statistics software (SPSS, Inc., Chicago, IL, USA).

## Results

### Culturing of hBMSCs

**Figure 1:**
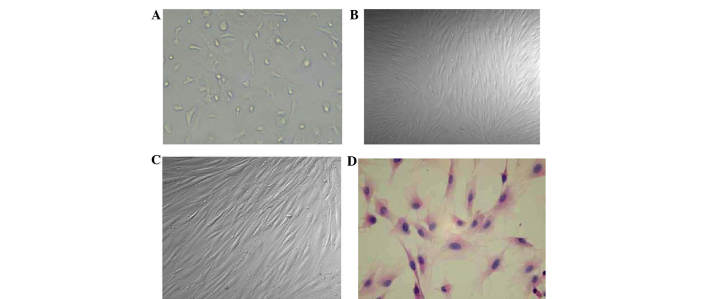
Cultured hBMSCs. (A) Cell morphology of hBMSC at initial plating. (B) Third generation of hBMSC cells are uniform with spindle shapes and a fibroblast-like morphology. (C) Sixth generation of hBMSC cells become more fusiform and evenly distributed. (D) Second generation of hBMSC cells were stained with hematoxylin and eosin (H&E). Clear nuclei with large nucleoli were observed. hBMSCs, human bone marrow stromal cells.

hBMSC cultures are shown in Fig. 1. The BMSC cell morphology at initial plating, the third passage, and the sixth passage are shown in Fig. 1A-C, respectively. A representative hematoxylin and eosin (H&E)-stained hBMSC sample is shown in Fig. 1D.

### Histological study

**Figure 2:**
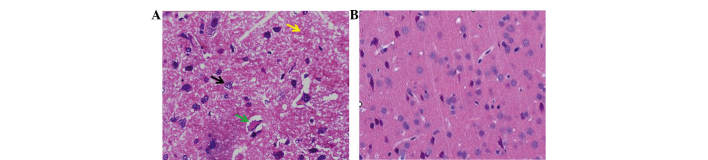
Hematoxylin and eosin (H&E) staining of brain tissue sections of rat models of cerebral ischemia. (A) Center of the infarction area in group C, which did not receive transplantation following ischemia/reperfusion (magnification, ×400). Staining was lighter in the infarction area compared with that in normal brain tissue due to the presence of vacuolation and mesenchymal edema (indicated by yellow arrow). The nuclei were small (indicated by black arrow) and deformed with cracked cytoblasts (indicated by green arrow). (B) Adjacent normal part of corresponding tissue in group C (magnification, ×400).

The histology of the infarction area in the rats from the three groups that underwent MCAO is shown in Fig. 2. The microscopy image shows that neurons had large cytoblasts with clear nucleoli and abundant cytoplasm. In the center of the ischemic region in MCAO rats, the staining was light with loose tissue and mesenchymal edema (Fig. 2A). The number of cells was decreased. Cells were swollen and deformed with fractured cytoblasts. Aberrations in the ischemic periphery were reduced, and there were few infiltrating inflammatory and glial cells. Fig. 2A shows the center of the infarction area in group C, which did not receive transplantation following ischemia/reperfusion. The staining was lighter in the infarction area compared with the normal brain with loose tissue and mesenchymal edema (indicated by yellow arrow). The number of cells was decreased. Cells were swollen (indicated by black arrow) and deformed with cracked cytoblasts (indicated by green arrow). Fig. 2B shows the adjacent normal section of the corresponding tissue in group C.

### Behavioral testing

**Figure 3:**
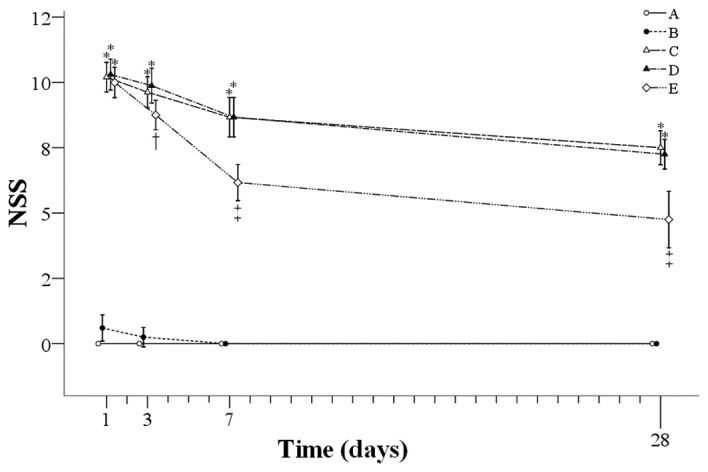
NSS in the five treatment groups. ^*^Significant difference compared with group A and B. ^†^Significant difference compared with groups A, B and D. ^‡^Significant difference compared with groups A-D. NSS, Neurological Severity Score.

The NSS for the five groups are shown in Fig. 3. The NSS in group A remained stable from day one to day 28. In group B, the NSS decreased from day one to day three and then remained stable from day seven to day 28. The NSS in the three groups that underwent reperfusion decreased from day one to day 28, among which group E showed the most significant decrease.

The differences between the groups at each time-point are summarized as follows: On day one, the NSS in the groups with reperfusion (groups C-E) were significantly higher than those in groups A and B, and similar results were observed on days three, seven, and day 28. In addition, the NSS in group E was significantly lower than that in group D on day three (8.75 versus 9.88), and the NSS in group E was significantly lower than that in groups C and D on both days seven and 28.

### VEGF

**Figure 4:**
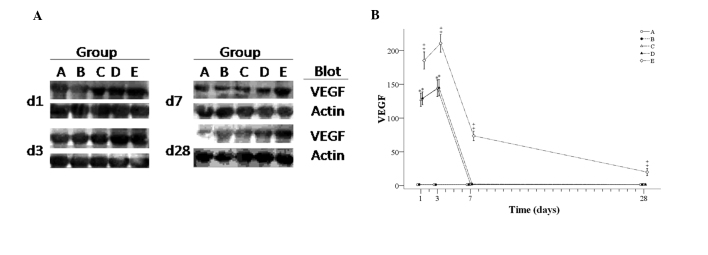
VEGF expression levels in the five groups. (A) Representative western blots for VEGF at the indicated time-points. (B) Number of VEGF-positive cells for the indicated time-points as determined by immunofluorescence staining. ^*^Significant difference compared with groups A and B. ^†^Significant difference compared with groups A, B and D. ^‡^Significant difference compared with groups A–D. VEGF, vascular endothelial growth factor; d, day.

Fig. 4A shows representative western blot images and Fig. 4B shows the number of VEGF-positive cells for each group during the experiment. The number of VEGF-positive cells in groups A and B remained stable from day one to day 28. In the other three groups with reperfusion, similar trends in the number of VEGF-positive cells were observed in groups C and D, but the number of VEGF-positive cells in group E was significantly higher than that in groups C and D from day one to day 28. Amounts of VEGF-positive cells initially increased from day one to day three, and steeply decreased to day seven, followed by stabilization in groups C and D. However, in group E, the levels increased from day one to day three and then continuously decreased. The differences between the groups at each time-point are summarized as follows: On days one and three, the number of VEGF-positive cells in the groups with reperfusion (groups C-E) was greater than that in groups A and B, and the number of VEGF-positive cells in group E was significantly higher than that in groups C and D. On days seven and 28, the number of VEGF-positive cells in group E was significantly higher that in the other four groups.

**Figure 5:**
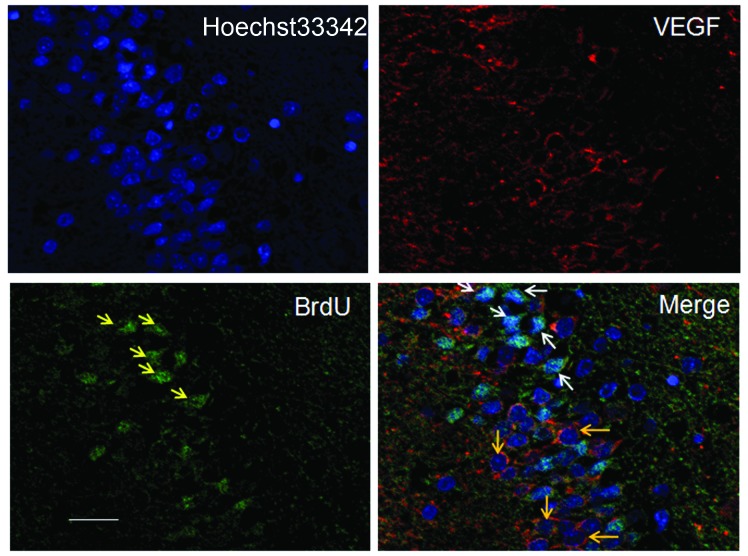
Immunostaining images of BrdU- and VEGF-positive cells seven days following transplantation of BrdU-labeled hBMSCs into the cortex of rat brains (bar, 20 μm). The cell nuclei are dark blue. VEGF is stained red and was expressed around the karyotheca or in the cytoplasm. The hBMSCs (yellow arrows in the non-merged image, white arrows in the merged image) were smaller than the neurocytes, with elliptical or round nuclei that were stained green due to BrdU labeling. Orange arrows point to VEGF-positive cells, which tended to have dark blue-stained nuclei, indicating a lack of BrdU staining and therefore, that these were original host cells. VEGF, vascular endothelial growth factor, hBMSCs, human bone marrow stromal cells. BrdU, 5-bromo-2-deoxyuridine.

Immunostaining images of BrdU- and VEGF-positive cells seven days following transplantation of BrdU-labeled hBMSCs into the cortex of rat brains are shown in Fig. 5. The cell nuclei were stained blue, whereas VEGF was stained red and was localized around the karyotheca or in the cytoplasm. The hBMSCs were smaller than the neurocytes, with elliptical or round nuclei, and they were stained green due to BrdU labeling.

## Discussion

In the present study, hBMSCs were injected into the brain of a rat model of MCAO following reperfusion. A histological study revealed that hBMSCs labeled with BrdU were predominantly located at the boundary between intact tissue and the infarction area. From day three the NSS of the rats that received BMSCs was significantly lower than the score of rats that underwent MCAO but did not receive BMSCs. From day one, the group that received BMSCs had significantly higher expression levels of VEGF than the other two groups that underwent MCAO. These results appear to support the hypothesis that the secretion of VEGF by BMSCs or the stimulation of host cells (rat cells) to secrete VEGF has an important role in the recovery from cerebral ischemia.

The present study appears to confirm the findings of previous studies in which BMSCs were transplanted into the rat cerebral ischemia model. Wakabayashi *et al* (9) found that rats injected with a human mesenchymal stem cell (MSC) line exerted a functional improvement compared with the controls and VEGF expression in the host cells was markedly increased, as well as epidermal growth factor (EGF) and bFGF expression. The only neurotrophic factor expressed by the MSCs appeared to be IGF-1. He *et al* (6) transplanted BMSCs and endothelial progenitor cells (EPCs) and found that the combination produced significantly greater functional improvement than BMSCs or EPCs transplanted alone. Furthermore, the BMSC/EPC group showed a significantly higher expression of VEGF, bFGF and BNDF. Bao *et al* (4) transplanted hBMSCs into rats with cerebral ischemia and found that following 14 days the rats that received hBMSCs had increased levels of VEGF, BDNF and NT-3. Wei *et al* (10) used hypoxia preconditioned BMSCs for transplantation. Hypoxia functionally improved BMSCs for transplantation, which may be due to an enhanced expression of trophic factors.

Compared with other cell types, BMSCs are good candidates for cell transplantation therapy. They are able to be obtained easily from patients or a bone marrow bank, and are able to be cultured and expanded in number with fewer ethical concerns than fetal stem cells. Furthermore, autologous transplantation of BMSCs or transplantation of BMSCs with the same human leukocyte antigen (HLA) subtype from a healthy donor may minimize the risk of rejection. For clinical application of cell transplantation therapy for the injured brain, transvenous or intrathecal cell injection is preferable as it is less invasive (20). However, the cell distribution into the ischemic brain is uncertain. Therefore, stereotaxic injection was used in the present study, and the results suggest that this method was successful. With the advancement of imaging technologies, the damaged tissue is able to be visualized easily. The present study suggests that the direct injection of cells has great potential in BMSC transplantation therapy for neurologic disorders.

Therapeutic angiogenesis is considered to be crucial for functional recovery following stroke. Angiogenesis requires a stimulus such as ischemia, tumor and inflammation. VEGF is known to be one of the most effective trophic factors that induces angiogenesis following stroke, which contributes to the recovery of the blood supply and functional recovery. In the present study, the hBMSC-injected group showed significant improvement in the score of the behavioral assessment tests compared with all the control groups. Findings of previous studies have shown hBMSC transplantation into the ischemic brain leads to improvement of the behavioral outcome (12,13,19). However, in these studies, the survival rate of BMSCs in the host brain was >10%, and the proportion showing neuronal differentiation was only 1–2%. The functional recovery in behavior following MSC transplantation is thought to be mediated by neurotrophic factors produced by BMSCs or by intrinsic parenchymal cells stimulated by BMSCs (21–23). The histological analysis in the present study revealed that the BrdU-labeled hBMSCs survived and migrated to the nearby intact tissue. The brains of BMSC-transplanted animals had a larger proportion of VEGF-positive cells, which may have contributed to the improvement in behavior. These results suggest that hBMSCs cultured *in vitro* followed by transplantation are able to survive and distribute widely following transplantation into the brain via stereotaxic injection. This procedure is effective for functional recovery from cerebral ischemia. Inducing the expression of VEGF may have an important role in this process.

Another explanation for the improved recovery following transplantation of hBMSCs is endogenous neurogenesis. Bao *et al* (4) reported that rats treated with hBMSCs showed enhanced endogenous cell proliferation in the hippocampal subventricular and subgranular zones, and an increased number of neuronal progenitor cells had migrated to the ischemic boundary zone from the subventricular zone and then differentiated into mature neurons with decreased levels of apoptosis. Neurogenesis and angiogenesis induced by trophic factors may have a role in the functional recovery following treatment with hBMSCs.

One limitation of the present study was that the sample size decreased at each assessed time-point. There were 10 animals in each group on day one, but only eight on day three, six on day seven and four on day 28. Another limitation was that the expression of only one trophic factor, VEGF, was assessed. Further studies are required to examine other potentially important underlying changes, including activation of VEGF signalling, phosphorylation of the VEGF receptor, as well as any changes in processes associated with angiogenesis and neurogenesis.

In conclusion, transplantation of hBMSCs into a rat model of MCAO appeared to improve neurologic recovery and increase the expression of VEGF in the ischemic region. VEGF may have an important role in functional recovery by stimulating angiogenesis. Transplantation of hBMSCs appears to be a promising stem cell therapy for treatment of acute stroke. Future investigations should focus on elucidating and confirming the mechanisms of the positive effect of transplantation of hBMSCs on functional recovery following cerebral ischemia.

## Figures and Tables

**Table I:** Neurological Severity Score.

Test	Points
Motor tests	
Raising rat by the tail	3
1 Flexion of forelimb	
1 Flexion of hindlimb	
1 Head moved >10° to vertical axis within 30 sec	
Placing rat on the floor (normal=0; maximum=3)	3
0 Normal walk	
1 Inability to walk straight	
2 Circling towards the paretic side	
3 Falling down to the paretic side	
Sensory tests	2
1 Placing test (visual and tactile test)	
2 Proprioceptive test (deep sensation, pushing the paw against the table edge to stimulate limb muscles)	
Beam balance tests (normal=0; maximum=6)	6
0 Balances with steady posture	
1 Grasps side of beam	
2 Hugs the beam and one limb falls down from the beam	
3 Hugs the beam and two limbs fall down from the beam, or spins on beam (>60 sec)	
4 Attempts to balance on the beam but falls off (>40 sec)	
5 Attempts to balance on the beam but falls off (>20 sec)	
6 Falls off: No attempt to balance or hang on to the beam (<20 sec)	
Reflexes absent and abnormal movements	4
1 Pinna reflex (shakes head when touching the auditory meatus)	
1 Corneal reflex (blinks with eye when lightly touching the cornea with cotton)	
1 Startle reflex (motor response to a brief noise from snapping a clipboard paper)	
1 Seizures, myoclonus, myodystony	
Maximum points	18

One point is awarded for the inability to perform the tasks or for the lack of an assessed reflex; 13–18, severe injury; 7–12, moderate injury; 1–6, mild injury.
